# 2-Arachidonoylglycerol Attenuates Myocardial Fibrosis in Diabetic Mice Via the TGF-β1/Smad Pathway

**DOI:** 10.1007/s10557-021-07307-7

**Published:** 2022-03-19

**Authors:** Zhengjie Chen, Liangyu Zheng, Gang Chen

**Affiliations:** Department of Anesthesiology, Sir Run Run Shaw Hospital, School of Medicine, Zhejiang University, Zhejiang, 310016 Hangzhou China

**Keywords:** Diabetic cardiomyopathy, 2-Arachidonoylglycerol, Myocardial Fibrosis, TGF-β1

## Abstract

**Purpose:**

Diabetic cardiomyopathy (DM) is the cause of late cardiac dysfunction in diabetic patients. Myocardial fibrosis is the main pathological mechanism, and it is associated with transforming growth factor-β1(TGF-β1) expression up-regulation. 2-Arachidonoylglycerol (2-AG) is an endogenous cannabinoid that can effectively improve myocardial cell energy metabolism and cardiac function. Here, we evaluated the protective effect of 2-AG on diabetic cardiomyopathy.

**Methods:**

Male C57BL/6 mice were injected with 2-AG intraperitoneally for 4 weeks (10 micro g/kg/day) after 12 weeks of diabetic modeling. After 4 weeks, heart function was evaluated by echocardiography. Heart structure was assessed by hematoxylin and eosin staining. Cardiac fibrosis was analyzed using immunohistochemistry, Sirius red stain, and western blot.

**Results:**

After modeling in diabetic mice, cardiac ultrasonography showed decreased cardiac function and pathological findings showed myocardial fibrosis. 2-AG could effectively inhibit the up-regulation of TGF-β1 and Smad2/3, reduce myocardial fibrosis, and ultimately improve cardiac function in diabetic mice.

**Conclusion:**

2-AG reduces cardiac fibrosis via the TGF-β1/Smad2/3 pathway and is a potential pathway for the treatment of cardiac dysfunction in diabetic mice.

## Introduction

As a globally prevalent disease, the number of patients with diabetes has doubled over the past 20 years [1]. Diabetic cardiomyopathy is defined as diffuse myocardial fibrosis and impaired systolic function in the absence of valvular disease, hypertension, and ischemic heart disease [2]. This fibrosis usually causes changes in the pumping and electrophysiological functions of the heart, which in turn induces heart failure and sudden cardiac arrest [3].

Collagen fiber is an important component of the extracellular matrix of cardiomyocytes, which supports the structure of the ventricle to maintain its geometry and function [4]. Long-term collagen deposition can lead to myocardial fibrosis and decreased ventricular compliance [4, 5]. In particular, the deposition of type I collagen fibers leads to ventricular rigidity [6]. Free fatty acid metabolism disturbances and hyperglycemia-induced collagen deposition promote the development of diabetic cardiomyopathy [5]. TGF-β1 regulates collagen deposition [7]. A selective increase in TGF-β1 in cardiomyocytes stimulated by high glucose triggers overexpression of the collagen-promoting gene by activating downstream Smad2/3 [8]. Furthermore, fibrosis of the myocardium during diabetes is exacerbated.

2-AG was the second endocannabinoid discovered [9]. It is able to regulate blood glucose and improve energy metabolism and is a potential diabetes treatment [10]. Endocannabinoids favor a protective role to the heart and its blood vessels [11]. Application of 2-AG in diabetic cardiomyopathy improves inflammation in cardiomyocytes [12]. Siegmund et al. [13] showed that 2-AG induces resistance to liver fibrosis, suggesting that 2-AG may have antifibrotic effects. However, whether 2-AG has this antifibrotic effect remains unclear in diabetic cardiomyopathy. In this study, we examined the effect of 2-AG in treating cardiac dysfunction in diabetic cardiomyopathy by relieving myocardial fibrosis and explored its mechanism.

## Materials and Methods

### Animals

Male C57BL/6 mice (20–22 g) were obtained from the Animal Experimental Center of Zhejiang University, and the mice were housed in a specific pathogen free (SPF) environment. All mice had free access to water and food.

The mice were randomly divided into the following groups: CON group, DM group, and DM + 2-AG group (nine per group). Mice in the DM and DM + 2-AG groups were fasted overnight and injected intraperitoneally with 100 mg/kg streptozotocin (STZ) (dissolved in 100 mM citrate buffer, pH 4.5, purchased from Sigma, USA). Mice in the CON group received an injection of the same volume of citrate buffer. On the 3rd and 7th day, two consecutive fasting (for 8 h) blood glucose measurements were obtained by the tail vein. Mice with 8 h fasting-blood glucose >11.1 mM were considered diabetic and continued feeding for 12 weeks. During the process of modeling diabetic cardiomyopathy, one mouse died of hyperglycemia in both the DM group and the DM + 2-AG group. After 12 weeks, the mice in the DM + 2-AG group were intraperitoneally injected with 2-AG (dissolved in physiological saline, purchased from Tocris, USA) at 10 micro g/kg/day for 4 consecutive weeks. The mice in the CON group and the DM group received the same dose of carrier for 4 weeks. The blood glucose and weight of all the mice were measured weekly during the 4 weeks. The cardiac function of the mice was measured after 16 weeks. Then killed by an overdose of 100 mg/kg ketamine hydrochloride (Ketanest, Pfizer, Germany) and 16 mg/kg xylazine hydrochloride (Rompun 2%, Bayer, Germany). The heart samples were weighed. The first third of the long axis of the entire heart near the apex of the mouse was immersed in paraformaldehyde to make paraffin sections. The remaining heart tissue was stored in liquid nitrogen for Western blot experiments. The serum was collected and stored in liquid nitrogen for detection of lipid levels.

### Echocardiographic Evaluation

Cardiac function was determined noninvasively by transthoracic echocardiography before death [14]. Electric heating pad was used for warming when mice were under anesthesia with isoflurane. During the echocardiography, one mouse died from an anesthesia accident. Doppler analysis was performed using a SONOS 5500 ultrasound (Philips Electronics, Amsterdam, The Netherlands) with a 15 MHz linear array ultrasound transducer to determine cardiac function. Left ventricle fractional shortening (LVFS) was calculated from left ventricular internal diameter at end-diastole (LVIDd) and internal diameter at end-systolic (LVIDs) using the equation (LVFS = [(LVIDd – LVIDs)/LVIDd] × 100). Left ventricle ejection fraction (LVEF) was calculated from left ventricle end-diastolic volume (LVEDV) and end-systolic volume (LVESV) using the equation of (LVEF = [(LVEDV – LVESV)/LVEDV] × 100).

### Detection of Serum Lipids

Blood samples were collected and centrifuged at 3000 rpm/min for 15 min at 4 °C to separate the supernatant (serum). The levels of TG, T-CHO, LDL-C, and HDL-C in the serum were measured according to the kit instructions (Nanjing Jiancheng, China).

### Histological Analysis

The heart tissue was fixed in 4% paraformaldehyde overnight, dehydrated and embedded in paraffin, sectioned at 5-μm thickness, and mounted on glass slides. After being de-waxed, the sections were stained with hematoxylin and eosin (H&E Assay Kit, Beyotime, China) and Sirius red (Sirius Heart Stain Kit, Solarbio, China), respectively. Images were observed and acquired using a Nikon microscope (Nikon, Japan). The H&E staining images use ImageJ image analysis software to calculate the average cross-sectional area of all cardiomyocytes in each field of view. The cross-sectional area of myocardial cells in six fields was randomly selected for each specimen. The Sirius red staining images use ImageJ image analysis software to perform semi-quantitative analysis by adjusting the gray value to distinguish between collagen fiber and non-collagen fiber areas [15]. For each slice, the positive area (stained in red) is identified in six random fields to measure the ratio between the positive area and the total image.

### Immunohistochemical Analysis

The sections were deparaffinized, rehydrated in gradient xylene and ethanol, antigen retrieval was performed by microwaving in 0.1 mol/L citrate buffer (pH 6.0), and the sections were then allowed to stand in a 3% hydrogen peroxide solution [16]. After blocking with 2.5% BSA (Sigma, USA), the sections were incubated with anti-collagen I (1:200, Abcam, UK) at 4 °C overnight and then secondary antibody (1:200, Santa Cruz, USA), colored by 3,3′-diaminobenzidine tetrahydrochloride (DAB; ZSGB-Bio, China), followed by sealing in neutral resin after dehydration in ethanol xylene. Images were taken under a Nikon microscope. The image analysis method is similar to Sirius red staining (the positive area stained in brown) [17].

### Western Blot Analysis

The myocardial tissue was homogenized and lysed in ice-cold RIPA buffer (150 mM sodium chloride, 0.1% sodium dodecyl sulphate (SDS), 0.5% sodium deoxycholate, 1.0% NP-40, and 50 mM Tris, pH 8.0), and the total protein concentration was quantified by using a BCA protein assay kit (Thermo, USA). A total of 30–50 μg of protein was loaded and separated by 10% SDS–polyacrylamide gel electrophoresis (PAGE). Then, the protein was transferred from the gel to a polyvinylidene fluoride membrane. After blocking with 5% skim milk and 0.05% Tween 20, the membrane was incubated overnight in primary antibody (TGF-β1(1:1000, Abcam:92486, UK), Smad2/3 (1:1000, CST:8685, USA), p-Smad2/3 (1:1000, CST:8828, USA), collagen I (1:1000, Abcam:34701, UK), and β-actin (1:1000, CST:4970, USA)). Bands were detected with a specific horseradish peroxidase-conjugated secondary antibody (CWBIO) (1:10000, Biosharp, China) and visualized by enhanced chemiluminescence reagents (Thermo, USA). Protein expression was quantified using ImageJ software.

### Statistical Analysis

All data are expressed as the mean ± standard deviation, and statistical analysis was performed using GraphPad Pro Prism 7.0. One-way ANOVA was used, and then multiple comparison tests with a Tukey correction were performed, with *P* < 0.05 considered a significant difference.

## Results

### Effect of 2-AG on Blood Glucose, Blood Lipids, and Body Weights of DM Mice

**Fig. 1:**
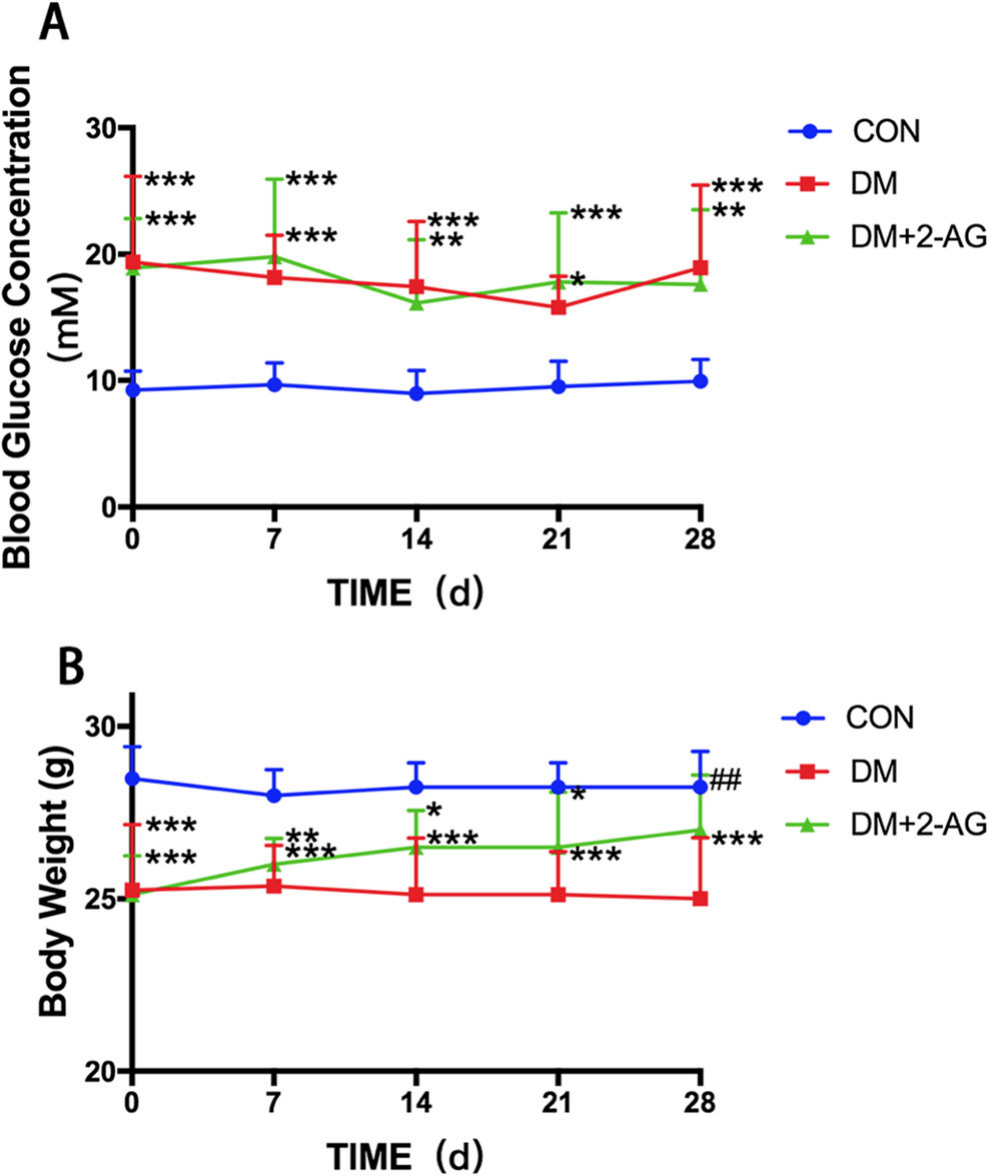
The effect of 2-AG on blood glucose and body weight. Blood glucose and weight were measured weekly during the study **A**. Blood glucose levels in the DM and DM + 2-AG groups were significantly higher than those in the CON group. **B**. Mice in the DM + 2-AG group had significantly improved DM-induced weight loss. ***P < 0.001, **P < 0.01, *P < 0.05 compared with the CON group; ##*P* < 0.01, compared with DM group. n = 8

In this study, after 12 weeks of diabetes modeling, 2-AG treatment was given for 4 weeks. Blood glucose and body weight were measured weekly during the study. Blood lipids were measured after serum collection. Compared with the levels in the CON group, the serum HDL-C levels in the DM and DM + 2-AG groups decreased significantly. There was no significant difference between DM group and DM + 2-AG group in serum HDL-C levels. Interestingly, the LDL-C level in the DM group was significantly higher than that in the CON group, and 2-AG reversed this trend. There was no significant difference between TC and TG among the groups (Table 1). There was a significant increase in blood glucose in the DM and DM + 2-AG groups, but there was no difference between the two groups (Fig. 1A). In addition, 2-AG treatment for 4 weeks improved DM-induced weight loss (Fig. 1B).

**Table 1:** Serum lipid levels

	CON	DM	DM + 2-AG	*P* value
HDL-C, mmol/L	1.94 ± 0.42	1.49 ± 0.29*	1.47 ± 0.25*	0.014
LDL-C, mmol/L	0.22 ± 0.02	0.33 ± 0.09*	0.20 ± 0.07^**##**^	0.008
TG, mmol/L	0.65 ± 0.14	0.70 ± 0.21	0.69 ± 0.08	0.794
T-CHO, mmol/L	1.68 ± 0.27	1.46 ± 0.27	1.60 ± 0.28	0.294

T-CHO, total cholesterol; TG, triglyceride; HDL-C, high density lipoprotein;

LDL-C, low density lipoprotein

*P < 0.05, compared with CON group; ^##^*P* < 0.01, compared with DM group. n = 8

### Effect of 2-AG on Cardiac Function and Ventricular Remodeling

**Fig. 2:**
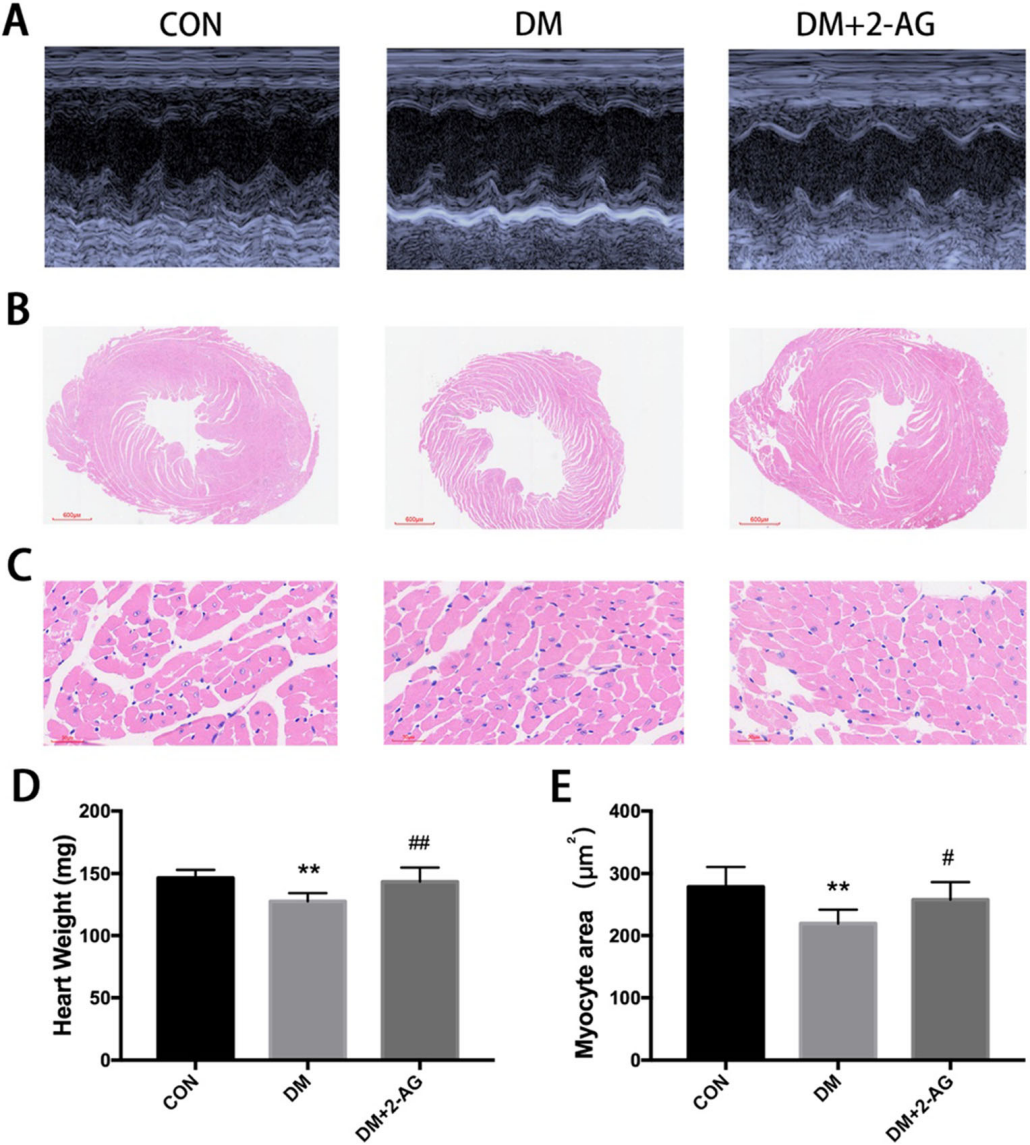
2-AG improves cardiac function and ventricular remodeling in diabetic mice **A**. Representative images of M-type echocardiograms. **B**. Overall view of H&E staining. Scale bar, 600 μm. **C**. Representative diagram of H&E staining cross section of the heart. Scale bar, 30 μm. **D**. Heart weight. E. Statistical graph of myocardial cross-sectional area. **P < 0.01, ***P < 0.001, compared with the CON group; #*P* < 0.05, ##*P* < 0.01, compared with the DM group. n = 7

To investigate whether 2-AG improves cardiac function in diabetic mice, we measured cardiac function by transthoracic echocardiography. As shown in Fig. 2A and Table 2, our results indicate that 2-AG protected mice from diabetes-induced cardiac functional deficits. LVEF and LVFS values of the mice in the DM group were lower than those in the CON group, while the values of these two indicators were significantly increased in the DM + 2-AG group compared with those of the DM group. The HR, FWd, FWs, PWd, PWs, and IVSd of DM mice was lower than that of the CON group. Treatment with 2-AG could simultaneously inhibit the reduction of HR, FWd, FWs, PWd, PWs, and IVSd. The overall view of the H&E staining heart cross-section also showed that the left ventricular cavity was significantly enlarged in the DM group, and the thickness of the left ventricular was reduced, while 2-AG reversed this effect (Fig. 2B). The cross-sectional area of myocardial cells in the DM group was significantly reduced compared with that of the CON group, and 2-AG improved the cross-sectional area of the myocardium caused by DM (Fig. 2C, E). In addition, we found that the heart weight in the DM group decreased significantly, and 2-AG reversed this effect (Fig. 2D).

**Table 2:** The effect of 2-AG on cardiac function in diabetic mice

	CON	DM	DM + 2-AG	*P* value
HR, bpm	515.57 ± 40.32	458.57 ± 32.44*	521.14 ± 60.97^**#**^	0.038
LVEF, %	80.97 ± 2.57	71.28 ± 6.72**	78.83 ± 4.99^**#**^	0.006
LVFS, %	43.66 ± 2.56	35.38 ± 5.34**	41.09 ± 4.96^**#**^	0.008
LVIDd, mm	3.50 ± 0.22	3.54 ± 0.30	3.48 ± 0.31	0.691
LVIDs, mm	1.97 ± 0.14	2.29 ± 0.24*	2.00 ± 0.27^**#**^	0.047
LVFWd, mm	0.84 ± 0.06	0.71 ± 0.04***	0.81 ± 0.07^**##**^	0.002
LVFWs, mm	1.01 ± 0.07	0.90 ± 0.12*	1.04 ± 0.10^**#**^	0.029
LVPWd, mm	0.86 ± 0.05	0.77 ± 0.05*	0.84 ± 0.08^**#**^	0.040
LVPWs, mm	1.07 ± 0.08	0.97 ± 0.08*	1.11 ± 0.11^**##**^	0.020
IVSd, mm	0.89 ± 0.04	0.81 ± 0.04*	0.89 ± 0.07^**#**^	0.021
IVSs, mm	1.10 ± 0.08	1.03 ± 0.08	1.13 ± 0.10	0.102

Transthoracic echocardiography was performed on control and diabetic mice at the conclusion of the study. Cardiac parameters: HR, heart rate; LVEF, left ventricular ejection fraction %; LVFS, left ventricular fractional shortening %; LVIDd and LVIDs, left ventricular internal diameter end-diastole and end-systole; LVFWd and LVFWs, left ventricular forward wall at end-diastole and end-systole; LVPWd and LVPWs, left ventricular posterior wall at end-diastole and end-systole; IVSd and IVSs, interventricular septal thickness at end-diastole and end-systole. *P < 0.05, **P < 0.01, ***P < 0.001 compared with CON group; #*P* < 0.05, ##*P* < 0.01 with DM group. n = 7

### Effect of 2-AG on Myocardial Fibrosis

**Fig. 3:**
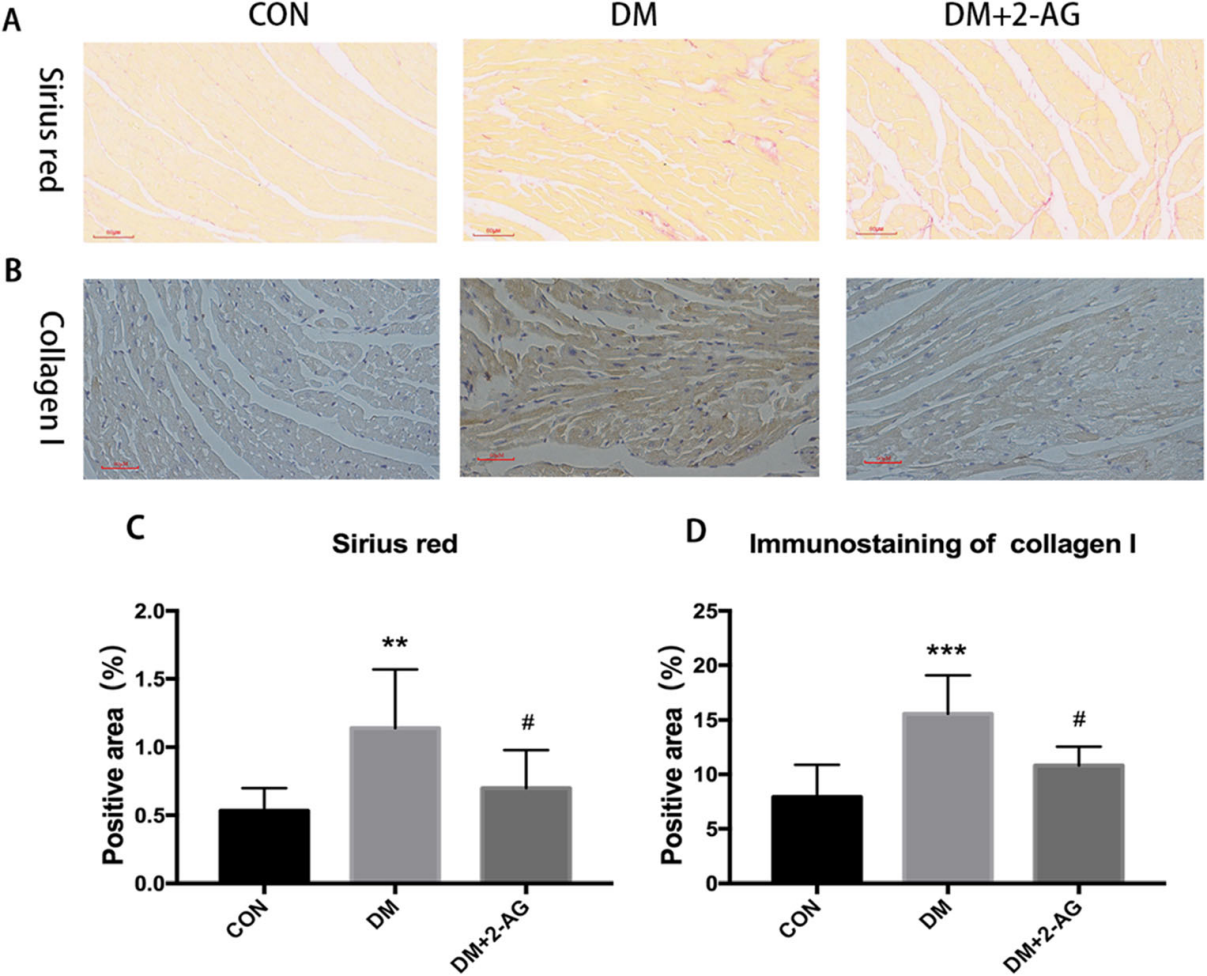
2-AG inhibits myocardial fibrosis and collagen deposition in diabetic mice **A**. Representation of the Sirius red-stained heart. Scale bar, 60 μm. **B**. A representative image of collagen I immunohistochemical staining. Scale bar, 60 μm. **C**. Statistical graph of the positive area of Sirius red staining. **D**. Statistical graph of immunohistochemical protein expression. **P < 0.01, ***P < 0.001 compared with the CON group; #*P* < 0.05 compared to the DM group. n = 7

To further explore the reasons for the improved heart function, we performed Sirius red staining. Sirius red staining showed an increase in the positive area in the DM group compared with that of the CON group, and 2-AG reversed this effect (Fig. 3A, C). To detect collagen I expression, immunohistochemistry staining assay was performed (Fig. 3B). The results showed that the heart tissue in the DM group had significant positive cells compared to that of the CON group, while 2-AG reduced this effect (Fig. 3D).

### Effect of 2-AG on the TGF-β Signaling Pathway

**Fig. 4:**
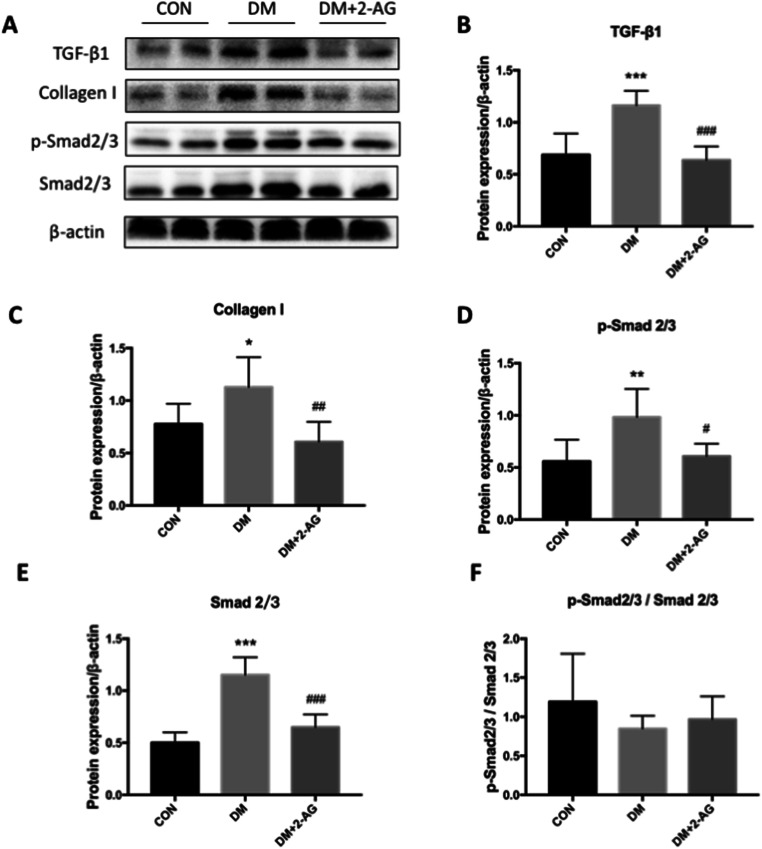
2-AG inhibits activation of the TGF-β1/Smad signaling pathway in myocardial fibrosis **A**. Western blot of TGF-β1, collagen I, p-Smad2/3, Smad2/3, and β-actin. **B**, **C**, **D**, and **E** are statistical representations of the relative expression of TGF-β1, collagen I, p-Smad2/3, and Smad2/3, respectively. F is the ratio of p-Smad2/3 and Smad2/3. *P < 0.05, **P < 0.01, ***P < 0.001 compared with the CON group; #*P* < 0.05, ##*P* < 0.01, ###*P* < 0.001, compared with the DM group. n = 3

As the TGF-β1/Smad2/3 pathway is a key factor in regulating fibrosis, we used western blot analysis to detect myocardial protein expression. As expected, the results showed that the total protein levels of TGF-β1, p-Smad2/3, and Smad2/3 in the lysate were significantly upregulated in the DM group (Fig. 4A, B, D, E), while 2-AG inhibited the expression of these proteins. Next, we measured the expression of collagen I (Fig. 4A). 2-AG downregulated collagen I expression, which was consistent with the immunohistochemistry results (Fig. 4C). We also calculated the ratio of p-Smad2/3 and Smad2/3. Because the total expression of p-Smad2/3 and Smad2/3 were both up-regulated, the ratio remained constant among the groups (Fig. 4F).

## Discussion

2-AG, a ligand for endocannabinoid receptors, has important protective effects in pathophysiological conditions such as shock and myocardial infarction [18]. Our study demonstrates that 2-AG can improve cardiac function in diabetic mice. H&E staining showed that 2-AG reversed ventricular remodeling in mice. Cardiac ultrasound showed that treatment with 2-AG improved left ventricular function decline in diabetic mice. This is related to 2-AG inhibiting the protein expression of TGF-β1/Smad2/3 and reducing myocardial fibrosis. Therefore, we first proposed that 2-AG reduces myocardial fibrosis in diabetic mice.

We use the type 2 diabetic mice model induced by high-fat feeding and low-dose STZ. Feeding mice with a high-fat diet with a fat content of 60% can cause contractile dysfunction and increase mortality after only feeding for 10 weeks [19]. When our diabetic mice were modeled for 16 weeks, the systolic function and the cross-sectional area of the myocardium were significantly reduced, indicating that the content and quality of the left ventricular myocardium decreased significantly. Nemoto et al. [20] showed similar results. After 12 weeks of diabetes, the mice were treated with 2-AG for 4 weeks. The systolic function and the cross-sectional area of the myocardium of diabetic mice in the treatment group increased significantly. Diabetic cardiomyocytes improve cytoplasmic Ca^2+^, especially mitochondrial Ca^2+^, which can enhance diastolic and systolic heart function [21]. 2-AG is synthesized and released on demand after an increase in the intracellular Ca^2+^ concentration produced by appropriate stimulation [22]. 2-AG reverses myocardial atrophy and may regulate calcium ions to improve myocardial function. Clinical studies have shown that diabetic cardiomyopathy is manifested as left ventricular hypertrophy associated with systolic/diastolic dysfunction and cardiac fibrosis in diabetic patients, which is an important factor leading to heart failure [23]. 2-AG is a potential drug for improving diabetic myocardial function. The endocannabinoid system has an impact on the central nervous system, cardiovascular diseases, diabetes, obesity, depression, and many other diseases [24]. The development of diabetic cardiomyopathy is a long process. 2-AG cannot achieve the purpose of treatment through short-term application. Whether long-term low-dose use of 2-AG will cause complications in humans or animals is unclear.

2-AG is an endogenous cannabinoid, which has the functions of regulating glucose and lipid metabolism [25]. Diabetes can cause myocardial lipid and carbohydrate metabolism disorders. Long-term insulin resistance can lead to a decrease in glucose metabolism in cardiomyocytes, but an increase in fat metabolism leads to a decrease in energy metabolism efficiency [26]. Our results indicate that 2-AG significantly reduces the diabetes induced elevation of serum LDL-C and appears to have no effect on blood glucose. Studies have shown that 2-AG can activate the AMPK signaling pathway in a CaMKKβ-dependent manner, reduce inflammation, and improve insulin sensitivity and glucose uptake [12]. Therefore, we believe that 2-AG increases heart weight and body weight by regulating body energy metabolism.

Myocardial fibrosis is important in the pathogenesis of diabetic ventricular remodeling and cardiac pump failure [27]. TGF-β1 is a key mediator in fibrosis [7]. Our research shows that the levels of TGF-β1, p-Smad2/3, Smad2/3, and collagen I protein in the myocardium of diabetic mice is increased. There is no significant difference in the ratio of p-Smad2/3 and Smad2/3. The increase in Smad2/3 phosphorylation may be due to the up-regulation of Smad2/3 expression rather than the up-regulation of activation itself. The activated Smad2/3 undergoes nuclear translocation, enters the nucleus, and directs the transcription and translation of collagen, and Smad7 negatively regulates the activation of the fiber gene [28, 29]. Increased collagen and increased cross-linking in the extracellular matrix lead to cardiac sclerosis, which causes changes in cardiac pump function [30]. Collagen I is a major contributor to this process [6]. Treatment with 2-AG reduced the upregulation of TGF-β1, and expression of the downstream proteins Smad2/3 and collagen I was also downregulated. Therefore, the potential mechanism of endogenous cannabinoid 2-AG on cardiomyocyte fibrosis in diabetic mice may be by reducing the expression of collagen I protein through the TGF-β1/Smad2/3 pathway, thereby reducing collagen deposition and reducing the degree of heart stiffness to improve ventricular function.

Our study showed that the endogenous cannabinoid agonist 2-AG is a potential drug for the treatment of diabetic myocardiac fibrosis and cardiac pump function damage. The downregulation of collagen I expression by inhibition of the TGF-β1/Smad2/3 pathway may be a potential mechanism. There are some limitations to this study. First, we did not set up the CON+2-AG group. Having the CON+2-AG will make our article more complete. The additional administration of 2-AG will not affect the cardiac function of the mice and will not cause harm to the mice [12, 22, 31]. Second, we should study in depth how 2-AG affects cardiometabolism. 2-AG works mainly through CB receptors [32]. Whether 2-AG affects heart fibrosis through CB receptors is unknown. In future research, we will continue to explore the mechanism of 2-AG on cardiometabolism and the safe dose range of long-term use of 2-AG.

## Data Availability

When necessary, raw data could be provided.

## Abbreviation

2-AG: 2-arachidonoylglycerol
DM: diabetic cardiomyopathy
HDL-C: high density lipoprotein
HR: heart rate
IVSd: interventricular septal thickness at end-diastole
IVSs: interventricular septal thickness at end-systole
LDL-C: low density lipoprotein
LVEF: left ventricular ejection fraction
LVFS: left ventricular fractional shortening
LVFWd: left ventricular forward wall at end-diastole
LVFWs: left ventricular forward wall at end-systole
LVIDd: left ventricular internal diameter end-diastole
LVIDs: left ventricular internal diameter end-systole
LVPWd: left ventricular posterior wall at end-diastole
LVPWs: left ventricular posterior wall at end-systole
SPF: specific pathogen free
STZ: streptozotocin
T-CHO: total cholesterol
TG: triglyceride
TGF-β1: transforming growth factor-β1

## Code Availability

Unavailable.
